# New geographic record in eastern Amazon Forest and potential distribution of *Amphidecta calliomma* (Lepidoptera: Nymphalidae)

**DOI:** 10.1002/ece3.9762

**Published:** 2023-02-02

**Authors:** Amanda Paracampo, Ulysses Madureira Maia, Vitor Hugo Freitas Gomes, Leonardo de Sousa Miranda, Tereza Cristina Giannini

**Affiliations:** ^1^ Instituto Tecnológico Vale Belém Brazil; ^2^ Programa de Pós‐graduação em Zoologia da Universidade Federal do Pará Belém Brazil; ^3^ Escola de Negócios Tecnologia e Inovação Centro Universitário do Pará Belém Brazil; ^4^ Lancaster Environment Centre Lancaster University Lancaster UK

**Keywords:** butterfly, environmental suitability, new occurrence, species distribution modeling

## Abstract

*Amphidecta calliomma* is a butterfly species that occurs in Colombia, Bolivia, Peru, Venezuela, Ecuador, Panama, and Brazil (in the states of Mato Grosso, Mato Grosso do Sul, Rondônia, and Pará). Here, we present a new occurrence of *A*. *calliomma* in the Carajás National Forest (Pará, eastern Amazon), expanding the known distribution of the species. We also provide species distribution model comparing the contribution of the new occurrence to species area of occurrence projections, supporting future field research. The projections reveal an expansion of area of occurrence for *A*. *calliomma* located mainly in the southeast portion of Amazon Forest. Despite its wide distribution, the small number of records of *A*. *calliomma* may indicate that the species has a low detectability in surveys. This study provides support for new surveys and reduces the knowledge gap about *A*. *calliomma*, thus supporting its conservation.

## Graphical abstract

We present a new occurrence of *Amphidecta calliomma* in the Carajás National Forest (Pará, eastern Amazonia) and determine potential areas of occurrence to support future field research. The record of *A*. *calliomma* for the Carajás National Forest is an important data for the distribution of the species, as it adds a significant percentage of the area of occurrence that can reach more than 3,000,000 km^2^.

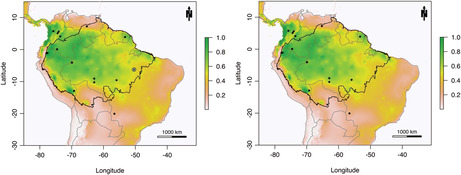

## 1 INTRODUCTION

Global declines in the abundance, richness, and distribution areas of insects have been reported in the last decades (Montgomery et al., 2020; Sánchez‐Bayo & Wyckhuys, 2019; Wagner et al., 2021). However, most of the declines are reported from geographically restricted studies, which makes it difficult to obtain reliable conclusions about the threats that affect species at different scales (Montgomery et al., 2020). Given the rapid loss of tropical forests each year (Curtis et al., 2018; Stokstad, 2018), there is an urgent need to collect data in tropical environments, where entomofauna are ecologically diverse and still little known (Stork, 2018). This can be achieved using species distribution modeling (SDM), which is a useful tool to address knowledge gaps about the distribution of species, based on species occurrences and environmental data (Peterson & Soberón, 2012).

Among the genera of Neotropical butterflies, *Amphidecta* Butler, 1867, has very little information available. To date, there are records in Colombia, Bolivia, Peru, Venezuela, Ecuador, Panama, French Guiana, and Brazil (in the states of Mato Grosso, Mato Grosso do Sul, Rondônia, and Pará) (D'Abrera, 1988; Lamas, 2004; Murray, 2000; Palo, 2017; Pardonnet et al., 2013; Radford et al., 2012; Ramos‐Artunduaga et al., 2021; Salazar, 2019; Silva et al., 2020; Sousa et al., 2019; Ureta, 1941). The species *Amphidecta calliomma* (C. Felder & R. Felder, 1862) shows a wide distribution over the Neotropic, but low number of occurrence records.

Here, we present a new occurrence record of *Amphidecta calliomma* in the eastern Amazon (Carajás National Forest; southwestern Pará), expanding the known distribution of this species. We also aim to determine potential areas of occurrence of this species to support future field research.

## 2 MATERIAL AND METHODS

### 2.1 Sampling

From November 05 to 14, 2019, we conducted a campaign to collect frugivorous butterflies in the Carajás National Forest (southwestern Pará state, Brazil). Butterflies were collected using Van Someren‐Rydon traps baited with a mixture of banana and beer (instead of sugarcane), which was fermented for 48 h, following methodologies adapted from Freitas et al. (2014) and Uehara‐Prado et al. (2005). The individuals captured in the traps were collected (SISBIO license number: 68977–1) and identified based on literature resources and with the help of the website “Butterflies of America” (https://www.butterfliesofamerica.com/L/Nymphalidae.htm, accessed in November 2020; Warren et al., 2013). After identification and preparation, the specimen of *A*. *calliomma* was incorporated into the entomological collection of the Museu Paraense Emílio Goeldi (MPEG.HLE 04045043; MPEG, Pará, Brazil).

### 2.2 Occurrence records

In addition to field collection, we retrieved data from Global Biodiversity Information Facility (GBIF, 2022; www.gbif.org, accessed in November 2022; DOI: https://doi.org/10.15468/dl.kgbph8) and SpeciesLink (https://specieslink.net/, accessed in November 2022) and from published articles, totaling 52 records. We also removed duplicate and nongeoreferenced data. We removed inconsistencies using a conservative pipeline (Gomes et al., 2018). Thus, our final database totaled 16 occurrence records (11 from the digital databases, 4 from articles, and 1 occurrence from our field collections; Table S1).

### 2.3 Climate information

We downloaded climate data with a resolution of 10 arc‐minutes (~18 km × 18 km) from the WorldClim database version 2.1 (www.worldclim.org, accessed in November 2022). We focused on noncorrelated climate data, based on ecological relevance. Butterflies are highly sensitive to climate as warm temperatures can stimulate their flight muscle efficiency and wind is a key component for flying animals and precipitation affects species richness (Checa et al., 2019; Turner et al., 1987). We downloaded and tested for correlation (coefficient threshold |ρ| < .7) seven historical climate variables: precipitation, water vapor pressure, solar radiation, wind speed, maximum temperature, minimum temperature, and average temperature.

### 2.4 Species distribution model

We used an algorithm based on maximum entropy (MaxEnt) to produce models of species potential distribution to estimate *A*. *calliomma* area of occurrence (AOO; Phillips et al., 2004; IUCN ‐ International Union for Conservation of Nature, 2022). We followed Gomes et al. (2019) and used background information to calibrate MaxEnt predictions based on data of tree species from Amazon Forest, since most of the occurrences of the *A*. *calliomma* are located in this biome. Background data are a sample from the study area used to characterize its environmental conditions (Phillips et al., 2009). Distribution modeling methods using background data generally outperformed those using presence–absence or pseudo‐absence information, especially when modeling mobile species (Fernandez et al., 2022). Also, background information methods are more flexible, producing more realistic and less overfitted predictions (Peterson et al., 2011). Since *A*. *calliomma* has little occurrence information available, we used a more flexible approach to understand the general distribution pattern of the species. We used *product*, *threshold*, and *hinge* features of MaxEnt (Boucher‐Lalonde et al., 2012; Merow et al., 2013). To evaluate the models, we used a null model approach (Raes & ter Steege, 2007). We tested the predictive performance of the *A*. *calliomma* models as estimated by the area under the ROC curve (AUC) against the predictive performance of 99 null models generated using the same number of occurrences of *A*. *calliomma* generated randomly. If the AUC of the models scores higher than the 95th best null models, this means that the chance of a model generated randomly showing a better performance is less than 5%. The models were converted in binary maps by using the 10th percentile training presence threshold, which omits the regions with environmental suitability lower than the lowest 10% of occurrence records (Gomes et al., 2018). We then clipped the binary maps by using the extent of occupancy (EOO) of the species plus a buffer of 300 km, based on the notion that the EOO is restricted by dispersal capabilities (De Ro et al., 2021; Gaston, 2009). We estimated *A*. *calliomma* AOO using the new occurrence sampled and comparing with the AOO estimation with no new occurrence. All calculations and analyses were performed with R version 3.6.3, including the R packages raster (Hijmans & van Etten, 2016), rgdal (Bivand et al., 2017), gstat (Pebesma & Heuvelink, 2016), dismo (Hijmans et al., 2016), rJava (Urbanek, 2017), and SDMTools (VanDerWal et al., 2019).

## 3 RESULTS

### 3.1 New occurrence

**FIGURE 1:**
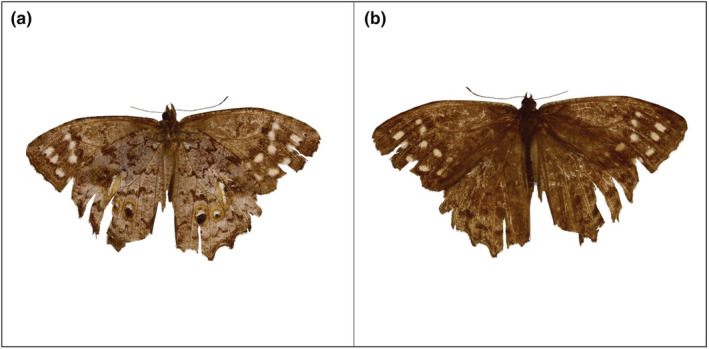
Specimen of *Amphidecta calliomma* collected in the Carajás National Forest. (a) Ventral view; (b) dorsal view. Photograph by Amanda Paracampo.

A specimen identified by AP as *A*. *calliomma* (Figure 1) was collected in the Carajás (at −6.2500, −50.4167) on November 11, 2019, in an understory trap. The new sampling point was placed in a deciduous forest, in which the height of the vegetation reaches up to 25 m. The individual had an anterior wing length of 30.35 mm, a body size of 20.14 mm, a proboscis length of 11.89 mm, an eye distance of 2.4 mm, camouflage colors and an eye patch with yellow rings.

A total of 16 occurrences were used to build the models when included the new occurrence (15 occurrences without it; Figure S1). We compared the results of modeling with and without the new occurrence point.

### 3.2 Species distribution model

The correlation test including the seven downloaded historical climate variables resulted on the selection of the four least correlated ones: precipitation, solar radiation, wind speed, and maximum temperature (Figure S2). Precipitation and wind speed were the most important environmental variables for our models, accounting for over 99% of contribution (Table S2). The model using the new occurrence of *A*. *calliomma* showed AUC of 0.789 and 0.799 excluding it. The 95th null model using the new occurrence scored an AUC of 0.7992, while 0.7859 excluding it. Both *A*. *calliomma* models showed higher AUC values than the 95th null models (Table S3).

**FIGURE 2:**
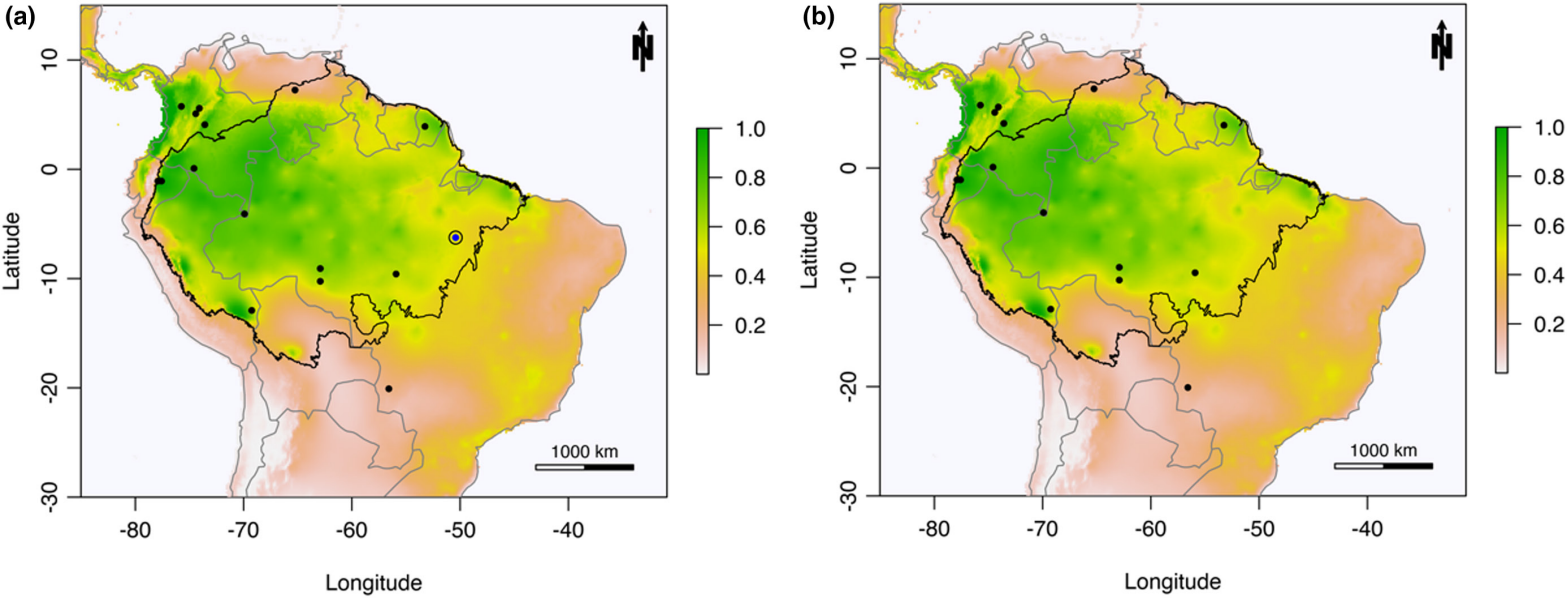
Potential distribution of *Amphidecta calliomma*. (a) Potential distribution including the new record. (b) Potential distribution excluding the new record. Black circles represent species occurrences. Blue dot with black circle is the new occurrence in the National Forest of Carajás. Black line represents the Amazon Forest border. Gray line represents the countries' border. Maps were created with custom R script (R CORE TEAM, 2019). Base map source (country.shp): ESRI (http://www.esri.com/data/basemaps, © Esri, DeLorme Publishing Company).

**FIGURE 3:**
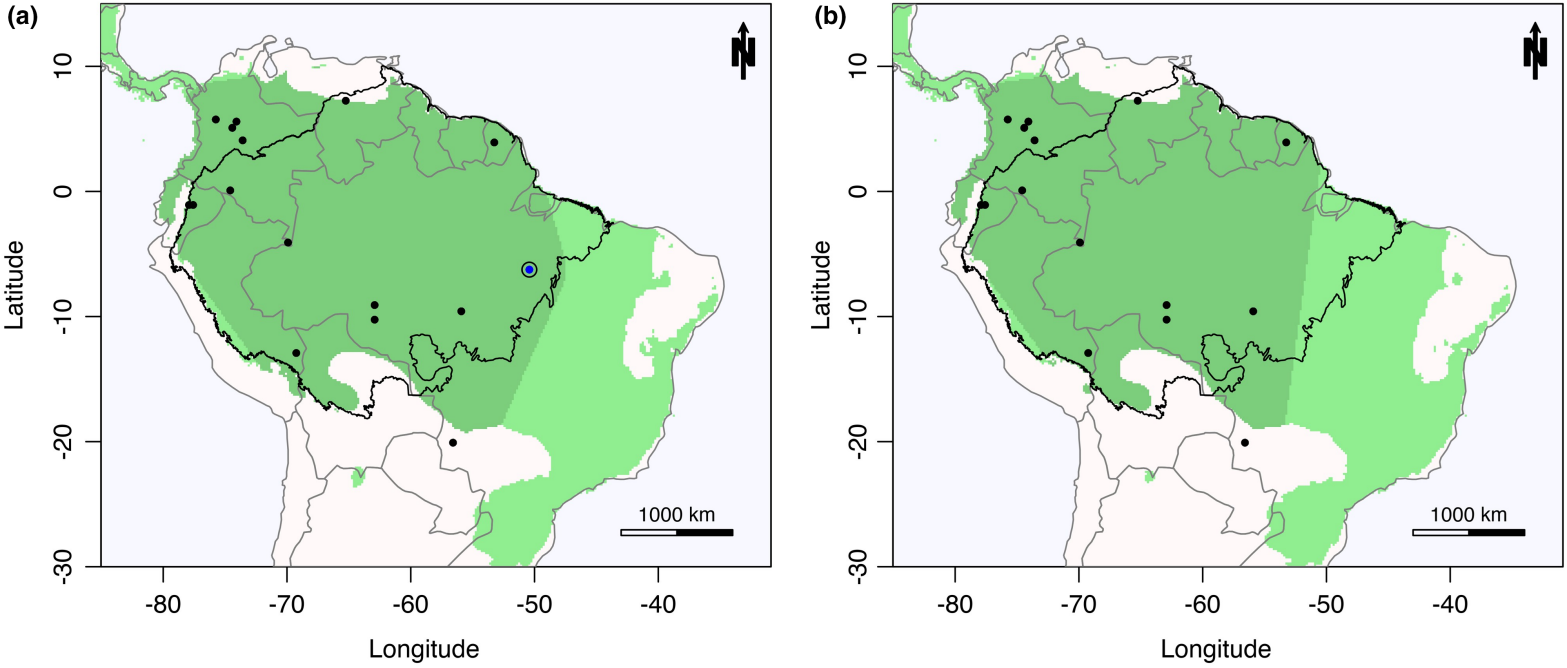
Area of occurrence for *Amphidecta calliomma*. (a) Area of occurrence including the new occurrence. (b) Area of occurrence excluding the new occurrence. Green color represents the area of occurrence cropped by the extent of occupancy plus a 300 k buffer. Light green color represents area of occurrence as predicted by the distribution models. Black dots are the species occurrences. Blue dot with black circle is the new occurrence in the National Forest of Carajás. Black line represents the Amazon Forest border. Gray line represents the countries' border. Maps were created with custom R script (R CORE TEAM, 2019). Base map source (country.shp): ESRI (http://www.esri.com/data/basemaps, © Esri, DeLorme Publishing Company).

The AOO revealed a relatively large area throughout the Amazon Forest, and also Brazilian Tropical Savannas and Atlantic Forest (Figure 2). The total AOO was 7,882,792 km^2^ with the new occurrence (Figure 2a), and 7,155,550 km^2^ excluding it, a reduction of 727,242 km^2^ (9% of the total) (Figure 2b). Considering the whole area beyond the EOO of the species, the total area was 11,189,146 km^2^ with the new occurrence and 10,970,806 km^2^ removing it (2% less). This shows a total 3,306,354 km^2^ of possible suitable areas for the species outside its extent of occurrence. Thus, according to the models, the AOO of the species is within the Amazon Forest biome (Figure 3). The new occurrence added little to the AOO of *A*. *calliomma* since the impact on its potential distribution as predicted by the models was small (Figure 3a). Only one record placed in the Central West part of Brazil was outside the AOO of *A*. *calliomma* (Figure 3b).

## 4 DISCUSSION

The high suitability values obtained by our model for central Amazon show a range that seems consistent for the species' distribution area, but our models also show a large area for Brazilian Tropical Savannas and Atlantic Forest. Although the new occurrence adds little information to the distribution modeling, it is important for understanding the current distribution of the species, showing also the significant role of protected areas for the conservation of *A*. *calliomma*. Therefore, it is essential that new occurrences would be recorded not only in the Amazon Forest but also outside its domain, to determine a more accurate distribution area.

Our predictive models also showed that *A*. *calliomma* can be distributed beyond the Amazon Forest, as far as the Atlantic Forest, expanding its potential distribution over roughly 3 million km^2^. However, there are no occurrence records of the species in the Atlantic Forest, which means that other factors, such as dispersion barriers, may restrict *A*. *calliomma* distribution further up to the Atlantic Forest.

Furthermore, the presence of *A*. *calliomma* in the eastern portion of the Amazon Forest within one of the few remaining pristine forests in the region is an important result, demonstrating the significant role of protected areas for the conservation of the species (Le Saout et al., 2013). Our study used only climate data to build an environmental suitability model, but other information can be included in distribution modeling to improve predictions, such as land‐use data and biotic interactions (Sobral‐Souza et al., 2021; Wisz et al., 2013). For example, the abundance of species of the subfamily Satyrinae is dependent on the distribution pattern of its host plants, since the larval stage of these butterflies usually develops in monocotyledons whose growth, abundance, and nutritional content are strongly seasonal (Sousa et al., 2019). However, there is still no information on the immature stages of *A*. *calliomma*, and consequently, there is no knowledge about its host plant. The only species within the genus for which a description of immature and host plants is available is *Amphidecta reynoldsi* Sharpe, 1890, which feeds on bamboo leaves (*Merostachys*) (*Poaceae*) (Freitas, 2004). Information on *Amphidecta pignerator A*. *Butler*, 1867, host plants is also lacking (DeVries, 1987). The *A*. *calliomma* specimen was collected in an area of seasonal semideciduous forest, which is characterized by seasonal leaf loss that occurs during the driest months, causing high light incidence. Amazon Semideciduous Forests are dominated by some genera, such as *Parapiptadenia*, *Cariniana*, *Lecythis*, *Tabebuia*, and *Astronium* (ICMBio, 2016), and the presence of bamboo leaves is uncertain.

In addition to these issues, the previous records of this species do not include precise information on collection sites, sampling efforts, or sampling methods. For example, in studies by Salazar (2019) and Silva et al. (2020), a specimen of *A*. *calliomma* was collected, but there is no description of which capture method was used. Murray (2000) also collected an individual of *A*. *calliomma* but did not report in which stratum the capture occurred. Thus, more detailed data on the habitats in which specimens were collected are useful to calibrate the species distribution model. Although our distribution model did not include information on land‐use and biotic interactions, we provided a primary evaluation of suitable areas for *A*. *calliomma* occurrence. This is the first step to help understanding the species distribution patterns, discovering populations and species limits, and developing strategies for species conservation. For future studies, we recommend new surveys, aiming at a more comprehensive description and evaluation of the ecological requirements of *A*. *calliomma*.

## 5 CONCLUSION

Our results contribute to the development of knowledge about the diversity of butterflies in the Amazon Forest and show that *A*. *calliomma* occurs further east in the biome than previously reported. Although the species potentially occurs in a wide area in the Amazon, few collections of the species were made in the region. Even collections made over long periods resulted in few specimens; this may suggest that there is a low abundance of this butterfly in nature, which is possibly an indication that this species is rare or that the species has low detectability or, when it is in the process of identification, it can be easily confused with other similar species. It is important to highlight that the objective of the predictive model presented in this work is not being a definitive map of the distribution of *A*. *calliomma*. It represents a first effort to point out search areas to refine the model in the future to obtain more records of occurrences and thus generate a more accurate distribution map with more refined models. Additional field studies in the areas highlighted in the present study are needed to broaden the knowledge of the biology of *A*. *calliomma* and subsequently improve occurrence models of this species. Knowledge about the species and its habitat represents an important step for conservation decision‐making, especially in the Amazon Forest, a biome under strong threat from deforestation and climate change.

## AUTHOR CONTRIBUTIONS

**Amanda Paracampo:** Conceptualization (equal); data curation (equal); formal analysis (equal); writing – original draft (equal). **Ulysses Madureira Maia:** Data curation (equal); formal analysis (equal). **Vitor Hugo Freitas Gomes:** Data curation (equal); formal analysis (equal). **Leonardo Sousa Miranda:** Conceptualization (equal); data curation (equal); formal analysis (equal). **Tereza Cristina Giannini:** Funding acquisition (equal).

## CONFLICT OF INTEREST

No potential conflict of interest is reported by the authors.

## Supporting information


Figure S1

Figure S2

Table S1

Table S2

Table S3

## Data Availability

All relevant data are within the manuscript and its Supporting Information files (two tables) are located in The Dryad Data Platform repository (https://doi.org/10.5061/dryad.mw6m9061d).
