# Supplementary figures and images for: New geographic record in eastern Amazon Forest and potential distribution of Amphidecta calliomma (Lepidoptera: Nymphalidae)
Source: Ecol Evol. 2023 Feb 2;13(2):e9762. doi: 10.1002/ece3.9762 (PMC9895319; doi:10.1002/ece3.9762)

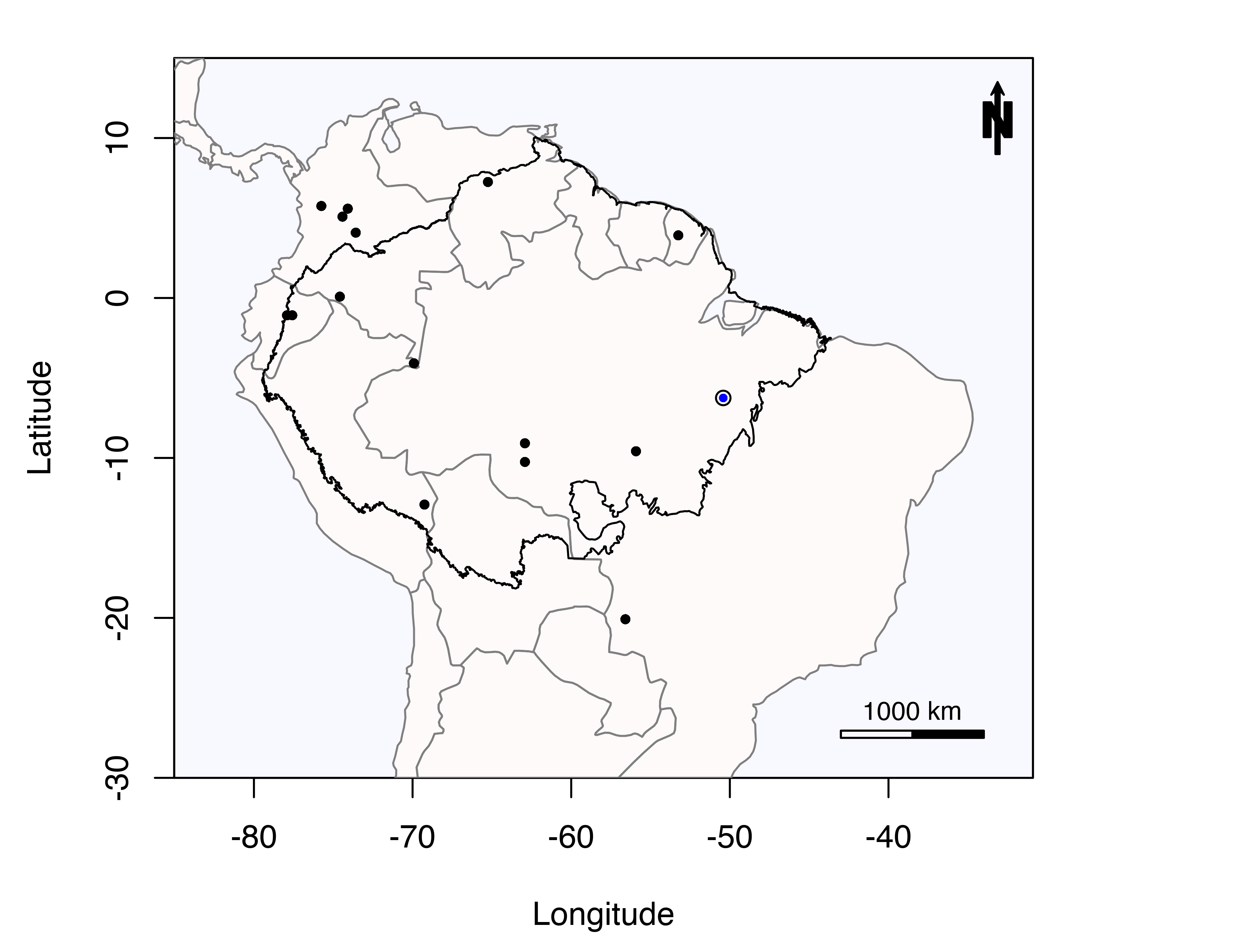

Supplement: Supplementary file 1 — Figure S1 [file ECE3-13-e9762-s001.tiff]

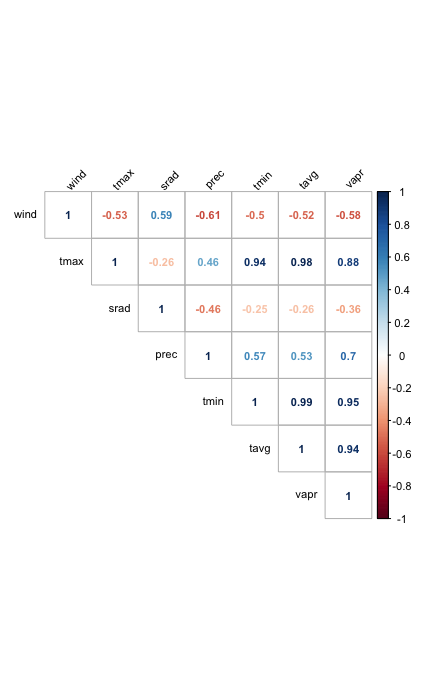

Supplement: Supplementary file 2 — Figure S2 [file ECE3-13-e9762-s004.tiff]
